# Randomized comparison of 4.5/6 Fr versus 6/7.5 Fr ureteroscopes for laser lithotripsy of lower/middle ureteral calculi: towards optimization of efficacy and safety of semirigid ureteroscopy

**DOI:** 10.1007/s00345-022-04173-2

**Published:** 2022-10-08

**Authors:** Mohamed Omar, Mohammed Dorrah, Ahmed Khalifa, Eid El Sherif, Khalid Sayedahmed, Yahya Ghazwani, Yasser A. Noureldin

**Affiliations:** 1 Department of Urology, Menoufia University, Shibin El Kom, Egypt; 2 Department of Urology, Rhein-Maas Hospital, Wuerselen, Germany; 3 Division of Urology, King Abdulaziz Medical City, Riyadh, Kingdom of Saudi Arabia; 4 Department of Urology, Faculty of Medicine, Benha University, Benha, Egypt

**Keywords:** Calculi, Ureteroscopy, Safety, Efficacy, Optimization

## Abstract

**Background and purpose:**

To compare 4.5/6 Fr versus 6/7.5 Fr semirigid ureteroscopes in terms of safety and efficacy in adult non-obese patients with middle or lower ureteric stones.

**Materials and methods:**

A total of 198 patients with middle/lower ureteric stone and a BMI ≤ 30 kg/m^2^ were recruited. Patients were randomized according to the size of ureteroscope into two groups: group 1 where a 4.5/6 Fr semi-rigid ureteroscope was used, and group 2 where a 6/7.5 Fr semi-rigid ureteroscope was used. Patient’s demographic, stone characteristics, intraoperative and postoperative outcomes including stone-free rate (SFR) and complications were compared.

**Results:**

Preoperative characteristics in terms of age, sex, BMI, and stone location, side, size, and HU were comparable between both groups (*p* values > 0.05). The overall SFR was significantly higher in group 1 (0.004). Balloon dilatation was not required in all patients of group-1 compared with 33% of group-2 (*p = *0.0001). The JJ stent was required in 10% of group-1 compared with 30% of group-2 (*p = *0.0004). Failure to reach the stone due to tight ureter occurred in 8% of group 2 (*p = *0.003), respectively. Traxer’s grade 1 ureteral injury occurred in 2% of group-1 versus 14% of group-2 (*p = *0.001). Consequently, hematuria was significantly lower in group-1 (1% vs. 8%; *p = *0.01), respectively. The hospital stay < 9 h was significantly higher in group 1 (*p = *0.0001).

**Conclusions:**

The 4.5/6 Fr semi-rigid Ureteroscope was associated with significantly higher SFR and shorter hospital stay, with lower ureteral injury, fewer double-J stenting, and without the need for intraoperative balloon dilatation for the ureter.

## Introduction

Management of ureteric stones has radically changed in the past 4 decades [1–3]. The main progress in ureteroscopic lithotripsy was after the introduction of Holmium lithotripsy which offered dusting and fragmentation of all ureteral stones with minimal complications [4]. According to the AUA (American Urological Association) guidelines, ureterscopy should be used as first-line therapy for patients with mid or distal ureteral stone [5].

Despite the versatility of flexible ureteroscopy (fURS), there is an economic and safety [6] consensus recommending the semi-rigid ureteroscopy (sURS) in the management of ureteric stones, especially for mid- and distal ureteral locations. Large caliber ureteroscopes provide more durability and better irrigation [7]. However, these scopes are associated with a higher need for ureteral dilatation and stenting, and increased complication rate [8] and the possibility of failure to reach the stone [9]. Some studies have reported a relation between high Body Mass Index (BMI) and increased stone-free rate (SFR) in lower ureteric stones [10], increased renal length [11] and uric acid stones [12].

This study aimed to compare the safety and efficacy of a 4.5/6 Fr versus 6/7.5 Fr ureteroscope in the treatment of middle and lower ureteric stones in non-obese patients.

## Materials and methods

### Study design

Between April 2016 and May 2019, patients at our tertiary care center presented with 1st episode of middle or lower ureteric radiopaque stones were prospectively randomized either to have laser lithotripsy with Wolf 4.5/6 Fr semi-rigid ureteroscope (Vernon Hills, Ill., USA) (group-1) or Storz 6/7.5 Fr semi-rigid ureteroscope (Tuttlingen, Germany) (group-2).

All patients were primarily evaluated with US and KUB, and those with suspected ureteral stones underwent a non-contrast CT (NCCT) for definitive diagnosis. This protocol was adopted to reduce radiation exposure. Laboratory tests including urine analysis, urine culture (UC), coagulation profile, and serum creatinine level were performed before surgery, and those with positive UC were treated with appropriate antibiotics according to culture and sensitivity. The study was performed under the ethical principles of the Declaration of Helsinki and approved by the local ethical committee of our institution. Informed consent was obtained from all patients.

**Fig. 1:**
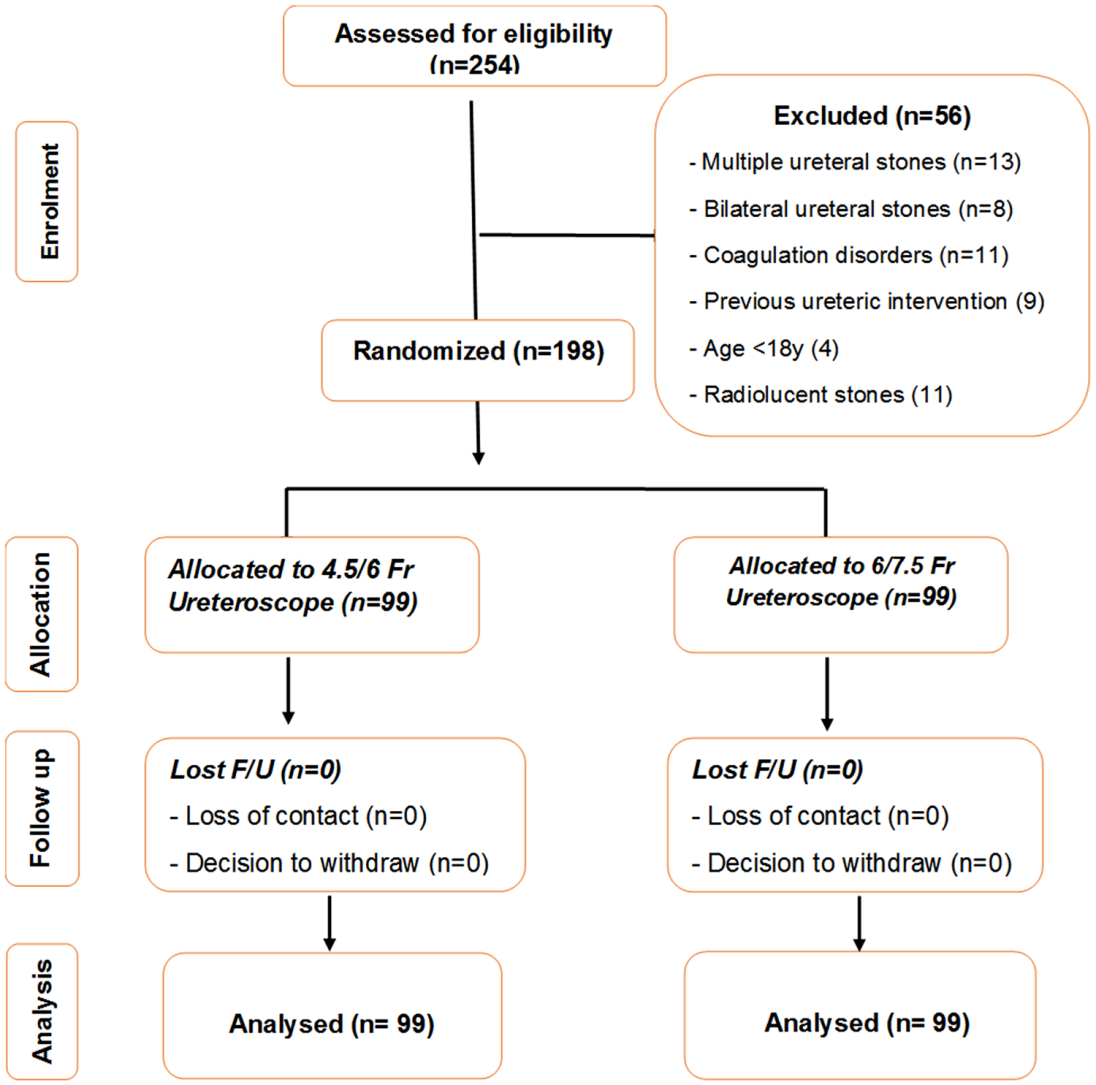
Flow chart

We excluded patients with multiple or bilateral ureteral stones, coagulation disorders, radiolucent stones, children (age < 18 years), and previous ureteric intervention including ureteroscopy or ureteric stent insertion (Fig. 1).

### Intervention

All procedures were performed under spinal anesthesia with the patients placed in the lithotomy position. A 272-µ laser fiber over Sphinx™ 30-Watt laser machine (Lisa Laser, Germany) and 1.9 F Zero tip basket was used. We used Sensor™ guidewire (Boston Scientific, USA) for initial cannulation of the ureter with other glide wires available on the surgery tray.

At the beginning of the procedure, the ureteroscope was inserted inside the urethra to the bladder followed by the insertion of a guidewire into the desired ureteric orifice under fluoroscopy guidance. Balloon dilatation (18Fr- 4 cm Uromax™, Boston Scientific, USA) was only used when the scope failed passively to advance inside the ureter. A Double-J (JJ) stent was inserted in selected cases at the end of the surgery, according to the use of ureteral dilatation or the presence of ureteral injury. In case of failure to reach the stones due to a tight ureter, a JJ stent was inserted for passive ureteral dilatation, and the procedure was aborted and done 2 weeks later. Patients were discharged on the same day once they were ambulant unless there was fever, significant hematuria " red or brown urine after being yellow" or agonizing pain.

### Data collection and outcome parameters

We collected patient’s demographics and stone characteristics. Intraoperative events were recorded including balloon dilatation, volume of irrigation fluid used (L), Holmium laser energy used (J), ureteral injury (classified according to Traxer’s classification) [6], failure to reach the stone due to tight ureter, the need for JJ stenting, and operative time. Additionally, postoperative outcomes in terms of SFR, the need for the auxiliary procedure, and postoperative complications (according to the Calvien-Dindo classification system) were reported. The SFR was assessed based on non-contrast CT (NCCT) 1-month postoperatively, with a cut-off value ≤ 3 mm residual fragment considered stone free. Our primary outcome was the SFR. Secondary outcomes included the incidence of complications in terms of ureteral injury, hematuria, and fever.

### Statistical analysis

Statistical analysis was performed with SPSS software, version 20.0 (IBM, Armonk, NY). Categorical variables were analyzed using the Chi-square test or the Fisher’s Exact test, and continuous variables were compared with the Student’s *t* test or the Mann–Whitney *U* test, whenever appropriate. A *p* value < 0.05 was considered significant. The sample size calculation was performed using the G* Power 3.1.9.2 for Windows. Uzun H and Akça N reported that the use of 4.5/6 Fr semirigid ureteroscopy improved the SFR to 97.5% compared with 72.7% with the use of and 6/7.5 Fr semirigid Ureteroscope [13]. Based on these expected proportions (P1/P2) between group-1 and group-2 for the primary outcome, a total sample size of 130 patients (65 in each group) was required with an (alpha error probability) of 0.05 and (1 − *β* error probability) of 0.95. The number was increased to 198 to correct for any drop in patients. Simple randomization, using the computer-generated random chart from https://www.randomizer.org/, that was printed and secured in closed envelopes with 1/1 allocation and patients were blinded to the procedure.

## Results

A total of 198 patients were recruited and randomized into 99 patients in group-1 (4.5/6 Fr semi-rigid ureteroscope) and 99 patients in group-2 (6/7.5 Fr semi-rigid ureteroscope). The mean stone size in group-1 was 10.8 ± 1.9 mm, with 40% in the middle ureter and 60% in the lower ureter; while the mean stone size in group-2 was 10.5 ± 2.1 mm, with 36% in the middle ureter and 64% in the lower ureter. There was no statistically significant difference between both groups regarding the demographics of patients and stones (Table 1).

**Table 1:** Baseline patient’s and stone characteristics

Variables	Group 1 (4.5/6 Fr URS) (99 patients)	Group 2 (6/7.5 Fr URS) (99 patients)	*p* value
Age (Year)	47 ± 3	47 ± 1	0.9
Gender			0.6
Male	64 (65%)	61 (62%)	
Female	35 (35%)	38 (38%)	
BMI (kg/m^2^)	23.3 ± 3.7	23.5 ± 3.4	0.4
Diabetes mellitus	8 (8%)	8 (8%)	0.99
Hypertension	10 (10%)	8 (8%)	0.6
Stone side			0.4
Right	51 (52%)	46 (46%)	
Left	48 (48%)	53 (54%)	
Stone location			0.5
Middle third	40 (40%)	36 (36%)	
Lower third	59 (60%)	63 (64%)	
Stone size (mm)	10.8 ± 1.9	10.5 ± 2.1	0.5
Hounsfield unit	803 ± 354	821 ± 347	0.7

All procedures were performed with a comparable mean operative time (OT); 37 ± 11 min and 34 ± 9.5 min, and a comparable mean irrigation fluid volume 16.3 ± 5.2 L and 18 ± 5.8 L in group-1 and group-2 (*p = *0.1, *p = *0.039), respectively. Balloon dilation to the ureter was used in nearly one-third of cases in group 2, while it has not been used in group 1 (*p* < 0.0001). The mean laser energy was significantly higher in group 1 (*p = *0.03). Dormia basket usage was insignificant between both groups (*p = *0.2) (Table 2).

**Table 2:** Intraoperative outcomes (mean SD or number and percentage)

Variables	Group 1 (4.5/6 Fr URS) (99 patients)	Group 2 (6/7.5 Fr URS) (99 patients)	*p* value
Balloon dilatation	0 (0%)	32 (33%)	0.0001
Irrigation fluid (L)	8.1 ± 2.6	9 ± 2.9	0.039
Laser energy (J)	3250 ± 310	2890 ± 247	0.03
JJ stenting	10 (10%)	30 (30%)	0.0004
Failure to reach stone due to tight ureter	0	8 (8%)	0.003
Basket usage	34 (34%)	31 (31%)	0.2
Operative time (min)	37 ± 11	34 ± 9.5	0.1

The procedure was completed with the need for JJ stenting in only 10% of cases in group 1 compared with 30% of cases in group 2 (*p* < 0.0004). Failure to reach the stone due to a tight ureter was an outcome in 8% of cases in group 2 (*p = *0.003) (Table 2).

Post-operative outcomes were in favor of group-1 vs. Group-2 in terms of the incidence of hematuria (*p = *0.01), ureteral mucosal injury Traxer’s grade 1 (2% vs. 14%, *p = *0.001), and Traxer’s grade 3 (0% vs. 3%, *p = *0.04), and hospital stay < 9 h (*p = *0.001), and overall SFR (*p = *0.004) (Table 3). On logistic regression analysis, the use of the 4.5/6 Fr URS was the only significant factor associated with more than 3 times higher stone-free status (OR: 3.7; *p = *0.006) (Table 4).

**Table 3:** Post-operative outcomes

Variables	Group 1 (4.5/6 Fr URS) (99 patients)	Group 2 (6/7.5 Fr URS) (99 patients)	*p* value
Peri-operative complications (Clavien-Dindo grading system)			
Ureteral injury (Clavien IIIa)			
Traxer’s grade 1	2 (2%)	14 (14%)	0.001
Traxer’s grade 3	0 (0%)	3 (3%)	0.04
Hematuria (Clavien I)	1 (1%)	8 (8%)	0.01
Fever (Clavien I)	9 (9%)	10 (10%)	0.8
Hospital stays			
< 9 h	79 (80%)	43 (43%)	0.0001
> 18 h	20 (20%)	56 (57%)	
Stone free rate	94%	77%	0.004

**Table 4:** Multivariable analysis for factors affecting stone-free status

Variables	*B* coefficient (SE)	Odds ratios (95% CI)	*p* value
Age	− 0.01 (0.01)	–	0.20
Female gender	–	0.82 (0.33–1.98)	0.65
Hounsfield Unit	− 0.00 (0.00)	–	0.55
Stone size	0.10 (0.10)	–	0.30
Lower ureter location	–	1.75 (0.73–4.17)	0.20
Group-1 (4.5/6 Fr URS)	–	3.7 (1.4–9.7)	0.006

*SE* standard error; *CI* confidence interval

## Discussion

Ureteric stone removal represents an everyday practice for most urologists. The great competition between ureteroscopy and shock wave lithotripsy (SWL) [14], had been resolved by the introduction of laser lithotripsy [15]. In a survey by Omar et al. [16], It had been reported that patients concerned about the risk of complications are more prone to select SWL while patients who have previous URS experience choose URS. Optimizing ureteroscopy outcomes with diminished risk of complications [17] would help more patients incline towards ureteroscopy. We evaluated the safety and efficacy of ureteroscopy by smaller (4.5/6 Fr) versus a relatively larger caliber (6/7.5 Fr) semirigid ureteroscopes for the management of distal and middle radioopaque ureteral stones.

The normal diameter of the ureter in adults is approximately 3–4 mm [18]. To gain access in such a diameter with an 11.5F rigid ureteroscope, balloon dilatation was invented [19]. However, ureteral balloon dilatation resulted in some complications. Greene LF [20], as one of the pioneers in testing the ureteral dilatation effect, showed that recurrent wide dilatation of the ureter results in permanent hydronephrosis and hydroureter. Boddy and co-workers [21] analyzed the balloon dilators' effects on the mini-pig ureter at 24-h and 4-week and recommended abandoning the routine use of maximal ureteric dilatation after discovering renal obstruction in most of the cases. Moreover, other investigators [22], reported that rapid dilation was associated with severe ureteral trauma, while safe dilation should be slow over a 10-min duration with a flow rate of 0.5 cc/min. Selmy et al. [23] considered two-fold dilatation as a safe procedure.

Preminger [9] and Pardalidis [24] reported a 5–8% incidence of failure or perforation that necessitated either a stent or nephrostomy tube after balloon dilatation. The consensus of most surveyed urologists by Preminger et al. [25] showed that they do not trust to leave the ureter non-stented after balloon dilatation and prefer at least 1-week stenting after ureteral dilatation to avoid temporary renal obstruction and hydronephrosis [26]. Such reports, confirm that entering and leaving the ureter without a change in its diameter would be more physiologic with better outcomes.

Since 1995, reports [27] had confirmed the safety of small caliber semirigid ureteroscope (8/9.8 Fr) [28] over conventional rigid ones. In the following decade, 7.5Fr ureteroscope had got popularity over the 10Fr rigid ureteroscope. The safety and efficacy of semirigid 4.5 Fr ureteroscope have been well established in previous reports [29, 30] with reduced overall complications.

In this study, we compared the safety and efficacy of a smaller diameter of 4.5 Fr versus 6.5 Fr in non-obese patients with middle and lower ureteric radioopaque stones. Our results had shown more acceptable SFR in the small ureteroscope group in comparison to the large ureteroscope group (94% vs. 77%) explained by the decreased failure to reach the stone due to tight ureter (0 vs. 8%). Similarly, Uzun and Akc [14] reported failure to advance the larger ureteroscope in five patients and replaced it with ultra-thin URS during the operation due to the physiological ureteric narrowing that lead to the overall increase in stone-free rate (SFR) up to 97.5%.

In addition, the 4.5 Fr group proved to show avoidance of balloon dilatation (0 vs. 33%) and less post-operative stenting (10% vs. 30%). Dogan et al. [31], reported that orifice dilation was used more frequently needed in patients who underwent the procedure with the large (8Fr) ureteroscope, which was accompanied by a higher incidence of complications.

The direct outcome from balloon dilatation included more incidence of hematuria that was statistically significant and necessitated the insertion of a ureteric stent in 30% compared with only 10% in the 4.5 Fr URS group (*p = *0.0004). This was congruent with the results of Uzun and Akc [14] who reported a significantly lower need for postoperative JJ stenting in patients treated with the ultra-thin URS (21.2%) compared with patients treated with 7.5/9.5Fr URS (46.3%).

Our results showed an 8% failure to reach the stone when using the 6/7.5 Fr URS in comparison to the zero failure to reach the stone in the 4.5Fr URS group (*p = *0.003). Atar et al. [32] reported higher stone migration, ureter laceration, and inability to reach the stone when using the 7.5 Fr URS versus the 4.5 Fr URS in the treatment of ureteral stones in preschool-age children. Such preferable outcomes may push our expectations for guidelines to mandate a second small 4.5 Fr ureteroscope to be present in the operation room for ureteroscopic lithotripsy as an alternative in difficult cases such as the tight ureter, intramural stone, pediatric patients or those on anti-coagulant medications. Topaktas and co-investigators [30] reported an SFR of 96.9% in pediatric ureteral stones treated with the 4.5Fr URS and none of the patients underwent active ureteral dilatations or required ureteral stent insertion for the purpose of passive dilatation. Furthermore, Söylemez et al. [29] reported the efficiency of the 4.5 Fr URS in entering difficult ureters of adults achieving a 95% success rate in 43 patients included in the study. Although one study [33] didn’t show a difference in SFR among different ureteroscopes sizes, it confirmed the safety of the 4.5Fr URS in terms of the lower rate of hematuria and mucosal injury and the fewer need for balloon dilatation.

Despite being a level 1 evidence study it still has some limitations which could be addressed as follow: First, we did not have the percent of alpha-blocker administration across both groups. Second, the surgeon was not blinded to the type of ureteroscope used during the procedure.

## Conclusions

The use of a 4.5/6 Fr semi-rigid Ureteroscope for management of middle/ lower third ureteral calculi in non-obese patients was associated with significantly higher SFR and shorter hospital stay, with lower ureteral injury, fewer double-J stenting, and without the need for intraoperative balloon dilatation for the ureter. Other multi-center studies are required to expand on our results.

## Abbreviations

URS: Ureteroscopy
fURS: Flexible ureteroscopy
sURS: Semi-rigid ureteroscopy
Fr: French
SWL: Shock wave lithotripsy
SFR: Stone free rate
BMI: Body mass index
NCCT: Non-contrast C T
HTN: Hypertension
DM: Diabetes mellitus
JJ: Double J stent
HFU: Hounsfield unit
AUA: American urological association
